# Participation in household decision making and justification of wife beating: evidence from the 2018 Mali Demographic and Health Survey

**DOI:** 10.1093/inthealth/ihab008

**Published:** 2021-03-15

**Authors:** Abdul-Aziz Seidu, Selorm Dzantor, Francis Sambah, Bright Opoku Ahinkorah, Edward Kwabena Ameyaw

**Affiliations:** Department of Population and Health, Faculty of Social Sciences, College of Humanities and Legal Studies, University of Cape Coast, Ghana; College of Public Health, Medical and Veterinary Sciences, James Cook University, Townsville, Queensland, Australia; Africa Centre of Excellence in Coastal Resilience (ACECoR), Centre for Coastal Management, University of Cape Coast, Cape Coast, Ghana; Department of Health, Physical Education, and Recreation, University of Cape Coast, Cape Coast, Ghana; School of Public Health, Faculty of Health, University of Technology Sydney, NSW, Australia; School of Public Health, Faculty of Health, University of Technology Sydney, NSW, Australia

**Keywords:** household decision making, intimate partner violence, Mali, wife beating

## Abstract

**Background:**

We assessed the association between women's participation in household decision making and justification of wife beating among married women ages 15–49 y in Mali.

**Methods:**

We employed a cross-sectional study design among 7893 women of reproductive age involving a two-stage sampling technique using version 6 of the Mali Demographic and Health Survey (MDHS) data, which was conducted in 2018.

**Results:**

Approximately 37% participated in at least one household decision while 23.4% reported that they would not justify wife beating in any of the stated circumstances. Women who participated in at least one household decision had lower odds (adjusted odds ratio [AOR] 0.834 [confidence interval {CI} 0.744 to 0.935]) of justifying wife beating. With respect to the covariates, we found that women 45–49 y of age had lower odds of justifying wife beating compared with those ages 15–19 y (AOR 0.569 [CI 0.424 to 0.764]). Women with higher education (AOR 0.419 [CI 0.265 to 0.662]) and those whose husbands had secondary education (AOR 0.825 [CI 0.683 to 0.995]) had lower odds of justifying wife beating. Women who lived in urban areas were less likely to justify wife-beating (AOR 0.328 [CI 0.275 to 0.390]) compared with those who lived in rural areas.

**Conclusion:**

This study suggests that participation in household decision making is associated with a significantly lower rate of justifying wife beating in Mali. These results underscore the need for various interventions to empower women to increase women's participation in decision making to reduce justification of domestic violence.

## Introduction

Globally, intimate partner violence (IPV) is a major social problem^1–3^ that has various social and health consequences for women and their children. It has been estimated by the World Health Organization (WHO)^4^ that 37% of women who have ever married in the world suffer from IPV at some point in their life, including sexual, emotional and physical violence.

In many low- and middle-income countries (LMICs), including Mali, one common type of IPV that is often perpetuated by the commonly held norms and gender roles in society is wife beating.^5^ Wife beating, is defined as ‘a situation whereby physical punishment is inflicted by a husband to “correct” an erring wife’.^6,7^ In several parts of the world, wife beating is considered the husband's right and is socially and culturally accepted.^8–10^ For instance, studies in India by Jejeebhoy^8^ and Rao^9^ underscore the extent to which women accept domestic violence as an undisputed aspect of marriage. According to Jejeebhoy,^8^ 36–38% of women in Tamil Nadu and 42–48% of women in Utter Pradesh suffer beating from their spouse and three in four women justify wife beating as the right of the man to put a disobedient wife under control. Again, a comparative study of IPV and justification of wife beating in sub-Saharan Africa revealed that both men and women justify wife beating for reasons such as when a woman argues with her husband, neglects the children or leaves home without the husband’s permission.^10^ Despite the fact that the magnitude of occurrence varies across the globe, it is an undeniable fact that the phenomenon exists in every society.^7^ Although there are various global as well as national campaigns and strategies to reduce IPV and wife beating, the reality is that too often it is covered up or tacitly condoned.^11^

The acceptance of wife beating is high in many LMICs. For example, 28% of women in Bangladesh indicated that it is acceptable for a wife to be beaten.^7^ In Nepal, almost 30% of couples indicated that it is acceptable for a wife to be beaten under certain instances.^12^ More than half of all women in Zimbabwe (53%)^13^ and about 90% of women in Uganda^14^ believed that wife beating was justified in at least one of several scenarios that were described to them. Rani et al.^15^ also found that a greater proportion (75%) of women in seven African countries support wife beating under certain circumstances. In Mali, Hayes and van Baak^16^ found that more than a quarter of women of reproductive age reported physical abuse.

Research has shown that the level of equality in decision making can significantly affect the chances of IPV.^17–19^ Collins,^20^ for instance, argued that where unequal power relations exist and household hierarchy prevails, perpetrators of violence may need to use actual or implicit force, sanctions and violence to maintain this structure of hierarchy and inequality. Studies have found a high prevalence of IPV when the man dominates in household decision making.^18,21^ Other scholars have also found that when a woman made the household decisions, there was an increased use of violence by the male partner, potentially as a response to the man's feeling of powerlessness.^19,22^ But in Mali, a study by Hayes and van Baak^16^ found no relationship between household decision making and physical violence.

There are many theories to explain IPV and thus assist scholars to better understand the cause of violence against women and its acceptance.^23^ In situating the variables underpinning women’s participation in household decision making and justification of wife beating in Mali, the subculture of violence theory (SVT) and the resource theory (RT) proved efficacious in determining the balance of power and influence in household decision making and approval of IPV.^24^ According to the RT, resources available to both men and women has the tendency to alter the gravity and nature of violence among partners.^24^ For instance, Goode^2^^4^ posits that the imbalance in decision-making capacity and approval of IPV is due to socio-economic differentials (i.e. income and social status), where the partner with the lower resources is unable to utilize the resources to attain power. Proponents of the RT argue that accessibility of resources, particularly for women, could change the dependency rate between women and men and may reduce men's dominance in decision making and eventually augment women’s participation in decision making with respect to domestic matters.

In applying the RT to our study, we adduce that autonomous and financially reliant women have some leverage of protection against acceptance and justification of IPV. Therefore the unavailability of such resources will not only undermine women’s participation in household decision making, but incline them to spousal violence acceptance. Also, lower socio-economic status may condition men to approve spousal violence on the basis that they are the sole breadwinners and thus wield domestic power in decision making.

In explaining the SVT, Wolfgang and Ferracuti^25^ assert that the presence of a cluster of values of violence within the value system of a subculture grants subtle approval within the socializing structure. This suggests that people in a given subculture, through the immediate family, gradually admit that violence is part of the social values and norms. In applying the SVT to the present study, we argue that participation in household decision making and justification of IPV is measured by the sociodemographic variables associated with women’s approval and justification of spousal violence, especially against conjugal women.

Despite this evidence, none of these studies have assessed the association between women’s participation in household decision making and justification of wife beating. Based on the inconsistency in findings on the relationship between household decision making and IPV, it is worthwhile to determine whether household decision making plays a role in the justification of wife beating. Therefore this study sought to assess the association between participation in household decision making and justification of wife beating. The findings of such a study will contribute to the fight against IPV in the sense that understanding the association of wife-beating attitudes may offer important insights to curb the cultural acceptance and intergenerational transmission of wife beating and prevent further violence.^7,26^

## Methods

### Data source

The data supporting this study were obtained from the version 6 of the Mali Demographic and Health Survey (MDHS), which was conducted in 2018. Specifically, the women recode file was used for the study. The MDHS forms part of the Demographic and Health Surveys (DHS) Program. DHS aims at monitoring health indicators in >85 LMICs globally. The survey captures a wide range of information on sexual and domestic violence as well as maternal and child health issues. The study has a two-stage sampling design. At the first stage, 379 primary survey units (PSUs) or clusters (104 in urban and 275 in rural areas) were systematically drawn with a probability proportional to their size in households from the list of enumeration sections (ESs) established during the general census of population and housing conducted in 2009. A household mapping and enumeration operation in the clusters was organized to draw an updated list of households in each ES to be used as a basis for stage sampling. In the regions of Kidal, Gao and Timbuktu, the mapping and enumeration of households was carried out just a few days before the data collection for the main survey. In the rest of the regions, this operation was carried out well before the main survey, from 25 May to 8 July 2018. After this, they compiled an updated list of households of each ES, a sample of 35 households in the Kidal, Gao and Timbuktu regions and 26 households in all the other regions with a systematic draw with equal probability. In households selected for the survey, all women 15–49 y of age usually living in selected households or present the night before the survey were eligible to be surveyed. For the purpose of this study, we dropped observations with missing information for the variables included in the analysis, which left data for 7893 currently married women as our analytical sample.

### Study variables

#### Dependent variable

Justification of wife beating was the dependent variable for our study. It was derived from five questions. Specifically, female survey respondents were asked if they would justify domestic violence under these five circumstances: going out without telling her husband, neglecting the children, arguing with her husband, refusing to have sexual intercourse and burning the food. For each of these circumstances, responses were ‘yes’, ‘no’ and ‘don't know’. These were coded as no=0, yes=1 and don't know=8. For the purpose of the analysis, only women who provided confirmatory responses (either yes or no) were included in the study. Following the methodology employed by Alam et al.,^7^ if a respondent thought beating would be justified, she was assigned a score of 0, but if a respondent thought beating would not be justified, she was assigned a score of 1. The internal consistency among the five variables (i.e. five circumstances) was assessed with Cronbach's α and a value of 0.8166 was obtained. All five circumstances were used to generate the binary outcome variable: 1 if the respondent thought beatings were justified in any circumstance and 0 if the respondent thought beatings were not justified in any circumstance.

#### Explanatory variable

The main explanatory variable of the study is self-reported participation in household decision making. This was derived from the responses to three individual questions regarding who within the household makes decisions in three circumstances: own healthcare, major household purchases and visits to family or relatives. For each circumstance, the response categories were as follows: (a) respondent alone; (b) respondent and husband, partner jointly; (c) husband/partner alone; (d) someone else and (e) other. The category (e) was deleted since there were few responses to that category (0.003%). These variables were dichotomously coded to be full or partial participation, described in options (a) and (b) and assigned a score of 1, and no participation, described in options (c) and (d) and assigned a score of 0. The internal consistency among the three variables (i.e. three circumstances) was Cronbach's α=0.7479. The predictor variable is equal to 1 if the respondent participated in any of the decisions and 0 if the women did not participate in any of the decisions.

### Control variables

We included a number of control variables due to their association with either the outcome or predictor variables.^7^,^27–29^ These included current age, respondent's and husband's education, respondent's work status, respondent's religion, parity, place of residence, wealth status and exposure to mass media (radio, television and newspaper). In the DHS, wealth is a composite measure computed by combining data on a household's ownership of carefully identified assets including a television and bicycle, materials used for house construction, sanitation facilities and type of water access. Principal component analysis was used to transform these variables into a wealth index by placing individual households on a continuous measure of relative wealth. The DHS segregates households into five wealth quintiles: poorest, poorer, middle, richer and richest. Some of these variables were recoded for easy interpretation and analysis. Religion was recoded as Christian, Islam and other. Parity was recoded (0, 1, 2, 3 and ≥4) and occupation was recoded as working and not working.

### Statistical analyses

The data were analysed using Stata version 14.2 for MacOS (StataCorp, College Station, TX, USA). Our analysis began with a descriptive investigation into the key sociodemographic characteristics and their relationship to justification of domestic violence. We then conducted a χ^2^ test to ascertain the relationship between participation in household decision making, sociodemographic characteristics and justification of sexual violence. Afterwards we conducted a χ^2^ test to ascertain the relationship between participation in household decision making, sociodemographic characteristics and justification of wife beating. This was done to identify significant variables to be considered for the inferential analysis. All these are reported in Table 1. At the inferential level, two binary logistic regression models were fitted. The first one (model I) accounted for only women's participation in household decision making and justification of wife beating, while the second (model II) controlled for the effect of the significant sociodemographic variables. Results for model I were presented as crude odds ratio (CORs) while adjusted odds ratios (AORs) were reported for model II with their respective confidence intervals (CIs) at a 5% margin of error. All analyses were performed considering the probability sample design. The svy commands were used in descriptive and bivariate analyses and probability weight, proposed by Rabe-Hesketh and Skrondal,^30^ was applied to the binary logistic regression analysis.

**Table 1.** Characteristics of study participants and percentage of females who do not justify wife beating, by sociodemographic characteristics

Variables	Weighted frequency	Weighted percentage
Age (years)		
15–19	791	10.0
20–24	1421	18.0
25–29	1726	21.9
30–34	1420	18.0
35–39	1222	15.5
40–44	773	9.8
45–49	540	6.8
Education		
None	5769	73.1
Primary	941	11.9
Secondary	1067	13.5
Higher	115	1.5
Husband's education		
None	5835	73.9
Primary	737	9.3
Secondary	980	12.4
Higher	341	4.3
Religion		
Christian	213	2.7
Islam	7403	93.8
Other	277	3.5
Wealth quintile		
Poor	1514	19.2
Middle	1622	20.6
Rich	1635	20.7
Richer	1630	20.7
Richest	1492	18.9
Occupation		
Not working	3104	39.3
Working	4789	60.7
Residence		
Rural	6196	78.5
Urban	1697	21.5
Parity		
0	593	7.5
1	1052	13.3
2	1104	14.0
3	1037	13.1
≥4	4107	52.0
Mass media exposure		
Frequency of reading Newspaper or magazine		
Not at all	7468	94.6
Less than once a week	250	3.2
At least once a week	175	2.2
Frequency of watching television		
Not at all	3073	38.9
Less than once a week	1695	21.5
At least once a week	3125	39.6
Frequency of listening to radio		
Not at all	2399	30.4
Less than once a week	1812	23.0
At least once a week	3682	46.7
Participation in household decision-making		
None	4993	63.3
At least one	2900	36.7

Source: 2018 MDHS.

### Ethical approval and consent to participate

The survey reported that ethical approval was granted by the Institutional Review Board of ICF International.^31^ Informed consent was sought from all the participants during the data collection exercise. We further obtained permission from the DHS Program for use of these data for the study.

## Results

### Descriptive results

**Figure 1.**
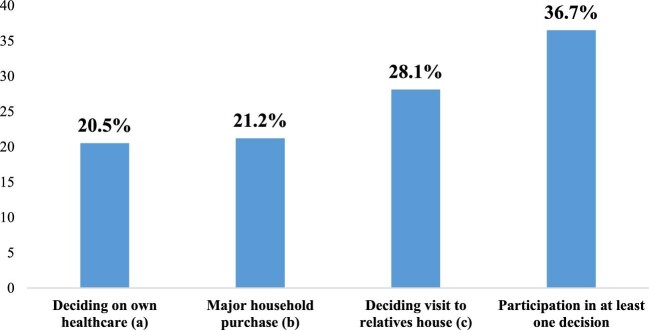
Percentage of female participation in household decision making. Source: 2018 MDHS.

**Figure 2.**
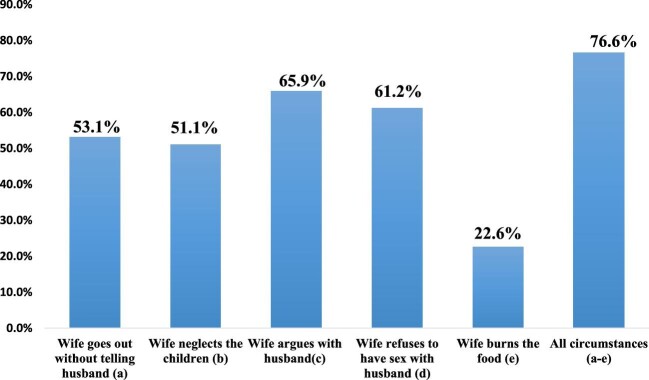
Percentage of females who would justify wife beating by circumstance. Source: 2018 MDHS.

Figure 1 shows the distribution of the percentage of females who reported participating in the various elements of household decision making. Approximately 37% participated in at least one household decision. A total of 28% of the women reported participating in deciding whether to visit their relatives and 20.5% participated in deciding their own healthcare. Overall, 76.6% of the respondents reported that wife beating in any of the stated circumstances is justifiable. Approximately 23% indicated that wife beating is justified when the wife burns food (22.6%) and 65.9% indicated that wife beating is justified when a woman argues with her husband (see Figure 2).

Tables 1 and 2 present the descriptive results of the study on sociodemographic characteristics, household decision-making capacity and justification of wife beating. Most of the women who did not participate in any household decision (78.0%) indicated it is justifiable for a wife to be beaten under any circumstance. Results from the χ^2^ test showed participation in household decision making (χ^2^=15.6, p<0.001), age (χ^2^=12.7, p<0.05), education (χ^2^=41.0, p<0.001), partners’ education (χ^2^=41.0, p<0.01), religion (χ^2^=6.8, p<0.05), wealth quintile (χ^2^=67.8, p<0.001), occupation (χ^2^=136.8, p<0.001), residence (χ^2^=215.5, p<0.001), parity (χ^2^=79.2, p<0.001), frequency of watching television (χ^2^=62.3, p<0.001) and frequency of listening to radio (χ^2^=63.0, p< 0.001) had a statistically significant association with justification of wife beating (Table 2).

**Table 2.** Justification of wife beating by sociodemographic characteristics

	Beating wife justified		
Variables	Yes	No	χ^2^ (p-value)
Participation in household decision making			15.6(p<0.001)
None	78.0	22.0	
At least one	74.1	25.9	
Age (years)			12.7 (p<0.05)
15–19	75.4	24.6	
20–24	74.8	25.2	
25–29	77.1	23.0	
30–34	77.4	22.6	
35–39	79.5	20.5	
40–44	76.3	23.7	
45–49	73.4	26.6	
Education			41.0 (p<0.001)
None	77.2	22.8	
Primary	78.9	21.1	
Secondary	73.3	26.7	
Higher	53.8	46.2	
Husband's education			17.44 (p<0.01)
None	77.4	22.6	
Primary	77.7	22.3	
Secondary	73.4	26.6	
Higher	69.5	30.5	
Religion			6.8 (p<0.05)
Christian	7.7	22.3	
Islam	76.3	23.7	
Other	84.1	15.9	
Wealth quintile			67.8 (p<0.001)
Poor	72.7	27.3	
Middle	82.5	17.5	
Rich	77.9	22.1	
Richer	78.0	22.0	
Richest	71.5	28.5	
Occupation			136.8 (p<0.001)
Not working	70.6	29.4	
Working	81.8	18.2	
Residence			215.5 (p<0.001)
Rural	80.9	19.1	
Urban	65.2	34.8	
Parity			79.2 (p<0.001)
0	66.5	33.5	
1	75.3	24.7	
2	74.0	26.1	
3	73.4	26.6	
≥4	80.3	19.7	
Mass media exposure			1.3 (p=0.527)
Frequency of reading newspaper or magazine			
Not at all	76.7	23.3	
Less than once a week	74.8	25.2	
At least once a week	73.8	26.2	
Frequency of watching television			62.3 (p<0.001)
Not at all	72.8	27.2	
Less than once a week	82.9	17.1	
At least once a week	77.6	22.4	
Frequency of listening to radio			63.0 (p<0.001)
Not at all	71.1	28.9	
Less than once a week	80.7	19.3	
At least once a week	78.3	21.7	

Source: 2018 MDHS.

### Binary logistic regression on participation in household decision making and justifying wife beating

As indicated in Table 3, the analysis revealed that women who participated in at least one household decision making had lower odds (COR 0.807 [95% CI 0.725 to 0.898]) of justifying wife beating compared with those who did not participate in any decision, and this continued even after controlling for covariates (AOR 0.834 [95% CI 0.744 to 0.935]).

**Table 3.** Binary logistic regression model on participating in household decision making and justification of wife beating

Variables	Model I COR (95% CI)	Model II AOR (95% CI)
Participation in household decision making		
None	Ref	Ref
At least one	0.807*** (0.725 to 0.898)	0.834** (0.744 to 0.935)
Age (years)		
15–19		Ref
20–24		0.859 (0.692 to 1.067)
25–29		0.825 (0.652 to 1.044)
30–34		0.762* (0.592 to 0.980)
35–39		0.788 (0.603 to 1.029)
40–44		0.655** (0.495 to 0.868)
45–49		0.569*** (0.424 to 0.764)
Education		
None		Ref
Primary		1.043 (0.868 to 1.254)
Secondary		0.864 (0.709 to 1.053)
Higher		0.419*** (0.265 to 0.662)
Husband's education		
None		Ref
Primary		0.948 (0.776 to 1.159)
Secondary		0.825* (0.683 to 0.995)
Higher		0.891 (0.656 to 1.211)
Residence		
Rural		Ref
Urban		0.328*** (0.275 to 0.390)
Religion		
Christian		Ref
Islam		1.016 (0.692 to 1.493)
Other		1.233 (0.721 to 2.107)
Wealth quintile		
Poor		Ref
Middle		1.366*** (1.144 to 1.631)
Rich		1.010 (0.852 to 1.198)
Richer		1.630*** (1.341 to 1.981)
Richest		2.093*** (1.632 to 2.683)
Occupation		
Not working		Ref
Working		1.634*** (1.458 to 1.831)
Parity		
0		Ref
1		1.397** (1.120 to 1.743)
2		1.444** (1.149 to 1.814)
3		1.326* (1.041 to 1.689)
≥4		1.875*** (1.492 to 2.357)
Frequency of watching television		
Not at all		Ref
Less than once a week		1.524*** (1.285 to 1.807)
At least once a week		1.315*** (1.132 to 1.528)
Frequency of listening to radio		
Not at all		Ref
Less than once a week		1.552*** (1.326 to 1.818)
At least once a week		1.448*** (1.269 to 1.652)
R^2^	0.0018	0.1430
Akaike information criterion	8584.4	7442.7
N	7893	7893

Ref: reference.

*p<0.05, **p<0.01, ***p<0.001.

Source: 2018 MDHS.

With respect to the covariates, we found that women 45–49 y of age had lower odds of justifying wife beating compared with those ages 15–19 y (AOR 0.569 [95% CI 0.424 to 0.764]). Women with higher education (AOR 0.419 [95% CI 0.265 to 0.662]) and those whose husbands had a secondary education (AOR 0.825 [95% CI 0.683 to 0.995]) had lower odds of justifying wife beating. Women who lived in urban areas were less likely to justify wife beating (AOR 0.328 [95% CI 0.275 to 0.390]) compared with those who lived in rural areas. In contrast, women who lived in the highest quintile households (AOR 2.093 [95% CI 1.632 to 2.683]), those who were working (AOR 1.634 [95% CI 1.458 to 1.831]), those with parity of four or more (AOR 1.875 [95% CI 1.492 to 2.357]) and those who watched television or listened to radio had higher odds of justifying wife beating.

## Discussion

The focus of our study was to assess the role of women's participation in household decision making in the justification of wife beating using data from the 2018 Mali DHS. Our study found that women who participated in at least one household decision making had lower odds of justifying wife beating compared with those who did not participate in any decision making. As explained by Alam et al.,^7^ women's participation in household decision making is regarded as empowerment. As a result, women who participate in household decision making are able to practice their rights and freedoms in the household.^7^ Consistent with our finding, Alam et al.^7^ observed that women who participate in at least one household decision have low odds of justifying wife beating.

Naved and Persson^32^ and Faramarzi et al.^33^ further articulated that women who have decision-making capacity and participate in household decisions can resist oppression and will not tolerate dictatorship from their spouse. Also, women's tolerant attitudes^33–35^ and the patriarchy system, as largely practiced in Africa, strengthen the phenomenon of IPV and justification of wife beating.^36^ Our findings imply that to reduce the justification by women concerning wife beating, women at all levels could be encouraged and empowered to participate in household decision making. It has also been noted that culture plays a very important role in women's participation in household decision making. For instance, Munemo^37^ noted that cultural issues such as patriarchy and women’s participation in multiple household tasks hinder women's participation in decision making. On this score, it is not surprising that women in urban locations were less likely to justify wife beating, as cultural ties are stronger in rural locations.^38–41^ This finding suggests that interventions that can motivate urban residents to disapprove of wife beating may be unsuccessful in the rural locations. However, the type of religion a woman belongs to could also affect her decision-making prospects^42,43^ and undoubtedly these factors may have consequences in the justification of wife beating.

With respect to the covariates, we found that the odds of justifying wife beating generally decreased with older age. This corroborates the findings of some Ghanaian^44,45^ and Indian studies^46^ where older women had low odds of justifying wife beating. In Nepal and Turkey, older women also had low odds of justifying wife beating.^47^ According to Stickley et al.,^48^ Uthman et al.,^49^ Young and Li^50^ and Waltermaurer et al.,^51^ as women progress in age, their perception and attitudes towards IPV change. Plausibly, older women develop self-confidence, self-esteem and self-reliance to resist oppression and violence from a spouse. However, younger women may not have been exposed to the power dynamics within the domestic environment and accept and justify wife beating, as observed by Waltermaurer et al.^51^ among Georgian women <25 y of age. In contrast, Islam et al.^52^ found that women's age was independent of their ability to justify or condemn wife beating. Our finding suggests that to tackle wife beating and its justification among women, interventions (such as empowerment in its various forms) should target younger women, and older women should serve as role models and counsel younger women in household dynamics (i.e. negotiation, navigation, communication etc.) such that younger women will not give in to their male partners.

Another key finding was that women with higher education and those whose husbands had a secondary level of education had lower odds of justifying wife beating. Education serves as an enabler to empowerment and an avenue to autonomy^46^ and similar findings have been reported in a number of multicountry studies in sub-Saharan Africa.^18,44^ Similar observations have also been made in some country-specific studies in Ghana^45^, Malawi^53^, and Bangladesh^54^ where women with a higher education background reported less likelihood of endorsing IPV. Also, the richest women and those who were working had high odds of justifying wife beating. Persons from rich homes are likely to be able to afford a good education. However, a woman may be working and wealthier, but these do not guarantee that the woman will be unaccepting of violent acts such as wife beating.^41,55^

Women who had high parity had high odds of justifying wife beating. Generally, women who jointly raise children with their partners have a higher risk of suffering from and accepting violence.^39^ It is probable that women who have more children are jointly taking care of these children with their partners/husbands. If so, they may be more compliant to wife beating as a way of subjecting themselves to the authority of their partners/husbands so as to guarantee consistency in sustenance for themselves and the children.^56^

Women who watched television at least once a week and those who listened to radio at least once a week had high odds of justifying wife beating. This contrasts with earlier findings from other sub-Saharan Africa countries.^39,40^ The critical role of mass media, especially radio and television, in reaching out to a large audience within sub-Saharan Africa is in no doubt.^57,58^ It is therefore possible that there has not been conscious and well-fashioned involvement of the media in anti-domestic violence campaigns in Mali. A cost-effective mass media experiment by Innovations for Poverty Action to reduce violence against women yielded a positive outcome in Uganda, and this may be an option for Mali as well.^59^

### Strength and limitations

The usefulness of our findings and their interpretation should be taken in light of some limitations. The study is limited by its cross-sectional nature and thus causal inferences cannot be drawn. Also, social desirability bias could affect the findings and may make the women underreport their justification of wife beating. Also, the use of a quantitative approach somewhat limits the findings. We therefore suggest the use of a qualitative paradigm in future research to ascertain which sociocultural determinants influence women's justification of wife beating in Mali. Despite these shortcomings, the study has revealed some compelling findings. The nationwide nature and the representativeness of the sampling strategy enhance the study's generalizability and the large sample size helped to boost the rigour of the study.

## Conclusions

Our inquiry into household decision-making capacity and justification of wife beating in Mali has revealed that women who make at least one household decision have a lower chance of justifying wife beating. This was also the case for women of older age, those with higher education and urban residents. Conversely, the richest women, those working, women with high parity and those who had constant engagement with television and radio were inclined toward justification of wife beating. The findings imply that in order to mitigate justification of wife beating in Mali, women must be equipped with negotiation skills and core competencies required for them to be empowered to influence household decisions. Placing such measures in legislative instruments and having well-outlined sustainability structures can prolong their longevity and impact. There is also the need for a conscious effort to utilize television and radio to educate the entire populace about the need to condemn any violent advances, irrespective of geographic location, wealth, occupation or marital status.

## Data Availability

The dataset is freely available to the public at https://dhsprogram.com/data/dataset/Mali_Standard-DHS_2018.cfm?flag=0.
